# Crystal structure of bis­[*trans*-di­chlorido­bis(propane-1,3-di­amine-κ^2^ *N*,*N*′)chromium(III)] dichromate from synchrotron data

**DOI:** 10.1107/S2056989016012755

**Published:** 2016-08-12

**Authors:** Dohyun Moon, Keon Sang Ryoo, Jong-Ha Choi

**Affiliations:** a Pohang Accelerator Laboratory, POSTECH, Pohang 37673, Republic of Korea; b Department of Chemistry, Andong National University, Andong 36729, Republic of Korea

**Keywords:** crystal structure, propane-1,3-di­amine, chloride ligand, *trans–anti* conformation, chromium(III) complex, dichromate anion, hydrogen bonding, synchrotron radiation

## Abstract

The structure of the title compound, [CrCl_2_(tn)_2_]_2_[Cr_2_O_7_] (tn = propane-1,3-di­amine; C_3_H_10_N_2_), has been determined from synchrotron data. The asymmetric unit contains one Cr^III^ complex cation and half a [Cr_2_O_7_]^2−^ anion. In the complex cation, the Cr^III^ ion is coordinated by the four N atoms of two propane-1,3-di­amine (tn) ligands in the equatorial plane and by two Cl atoms in a *trans* configuration, displaying a distorted octa­hedral coordination sphere. The two six-membered rings in the complex cation have an *anti* chair–chair conformation with respect to each other. The mean Cr—N(tn) and Cr—Cl bond lengths are 2.09 (1) and 2.320 (2) Å, respectively. The slightly bent dichromate anion is disordered over two sets of sites (occupancy ratio = 0.7:0.3) and has a staggered conformation. The crystal structure is stabilized by inter­molecular hydrogen bonds involving the NH_2_ groups of the tn ligands as donors and the O atoms of the [Cr_2_O_7_]^2−^ anion and chlorido ligands as acceptors.

## Abstract 2

In the title organic–inorganic salt, [CrCl_2_(tn)_2_]_2_[Cr_2_O_7_] (tn is propane-1,3-di­amine), the Cr^III^ ions are coordinated by four N atoms from two tn ligands and two chloride ions in a *trans* geometry, displaying a distorted octa­hedral arrangement. The crystal packing is stabilized by N—H⋯Cl and N—H⋯O hydrogen bonds.

## Chemical context   

Propane-1,3-di­amine (tn) can act as a bidentate ligand to a central metal ion *via* its two nitro­gen atoms, forming a six-membered ring. The [Cr*L*_2_(tn)_2_]^+^ (*L* = monodentate ligand) cation can adopt either *trans* or *cis* geometric isomers. In addition, there are two possible conformations with respect to the six-membered rings in the *trans*-isomer. The carbon atoms of the two chelate rings of the tn ligands can be located on the same side (*syn* conformer) or on opposite side (*anti* conformer) of the equatorial plane (Choi *et al.*, 2012 ▸). The preference for *syn*- or *anti*-conformation in the complex cation is an area of current inter­est because infrared or electronic absorption spectroscopic methods are not useful in determining the *syn* or *anti* conformations of the six-membered chelate rings in these transition metal complexes. The different arrangements of the two six-membered chelate rings of the tn ligands may be dependent on the packing forces and counter-anions in the crystal structure.
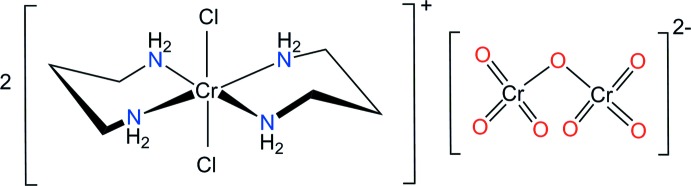


The shapes and sizes of counter-anions also play important roles in chemical, biological and environmental processes (Gadre *et al.*, 1992 ▸; Fabbrizzi & Poggi, 2013 ▸; Santos-Figueroa *et al.*, 2013 ▸). The dichromate ion is environmentally important due to its high toxicity and its use in industrial processes (Yusof & Malek, 2009 ▸; Goyal *et al.*, 2003 ▸). Here, we report on the synthesis and structure of [CrCl_2_(tn)_2_]_2_(Cr_2_O_7_), (I), in order to determine the conformations of the two six-membered chelate rings of the tn ligands and of the [Cr_2_O_7_]^2−^ anion.

## Structural commentary   

**Figure 1:**
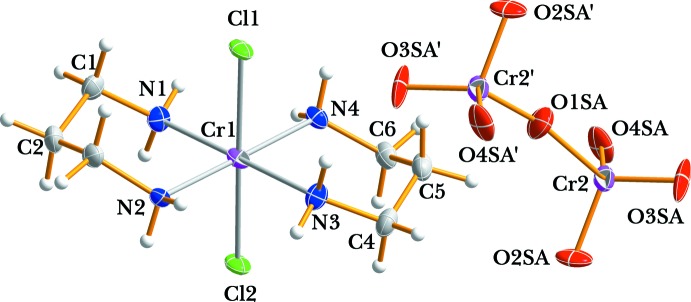
A perspective drawing of the complex cation and the anion with displacement ellipsoids at the 30% probability level. The primed atoms are related by symmetry code (−*x* + 2, −*y* + 1, −*z* + 1). Atoms of the minor disorder component have been omitted for clarity.

The structure of (I) shows another example of a *trans*-[CrCl_2_(tn)_2_]^+^ cation but with a different counter-anion (Kou *et al.*, 2001 ▸; Choi & Clegg, 2011 ▸; Moon *et al.*, 2012 ▸). The asymmetric unit comprises one Cr^III^ complex cation and half a [Cr_2_O_7_]^2−^ anion, the other half being completed by inversion symmetry. In the complex cation, the four nitro­gen atoms of the two tn ligands occupy the equatorial sites and two chlorine atoms coordinate to the Cr metal centre in a *trans* configuration. The Cr^III^ complex cation and the anion in the title compound are depicted in Fig. 1 ▸. The two six-membered rings involving the tn ligands have stable chair conformations. The two chelate rings in the Cr^III^ complex cation adopt the *anti* chair–chair conformation with respect to each other. The Cr—N(tn) bond lengths [range 2.0814 (19) to 2.1020 (19) Å] are in good agreement with the distances found in *trans*-[CrCl_2_(tn)_2_]ClO_4_ (Choi & Clegg, 2011 ▸) or *trans*-[CrCl_2_(tn)_2_]_2_ZnCl_4_ (Moon *et al.* 2012 ▸). As expected, the average Cr—Cl distance of 2.320 (2) Å is longer than that of Cr—F found in *trans*-[CrF_2_(tn)_2_]ClO_4_ (2.085 (4) Å; Vaughn & Rogers, 1985 ▸), and slightly shorter than of Cr—Br found in *trans*-[CrBr_2_(tn)_2_]ClO_4_ [2.4681 (4) Å; Choi *et al.*, 2012 ▸]. The bond angles of the two six-membered chelate rings around the Cr^III^ atom are 90.07 (8) and 91.25 (8)°. The other N—C and C—C bond lengths and Cr—N—C, N—C—C and C—C—C angles are also of usual values for tn ligands in chair conformations (Choi & Clegg, 2011 ▸; Moon *et al.*, 2012 ▸). The [Cr_2_O_7_]^2−^ counter-anion is positionally disordered and remains outside the coordination sphere of the Cr^III^ cation. It is of inter­est to compare the conformation of the [Cr_2_O_7_]^2−^ anion with that found in other ionic crystals. The [Cr_2_O_7_]^2−^ anion in compound (I) is in a staggered conformation, in contrast to that observed in K_2_Cr_2_O_7_. In the latter, two nearly tetra­hedral CrO_4_ groups are in an almost eclipsed conformation (Brandon & Brown, 1968 ▸), when viewed along the backbone of the dichromate anion. In (I), the O—Cr2—O bond angles of the major disordered component range from 102.3 (2) to 122.2 (8), while the terminal Cr2—O bond lengths vary from 1.554 (3) to 1.639 (4) Å, with a mean terminal Cr2—O bond length of 1.60 (4) Å. The bridging Cr2—O1*SA* bond has a length of 1.729 (15) Å, with a Cr2—O2*S*—Cr2 bond angle of 160.1 (4) Å. These values are comparable to those reported for [Cr(urea)_6_](Cr_2_O_7_)Br·H_2_O (Moon *et al.*, 2015 ▸). A further distortion of the anion is due to its involvement in hydrogen-bonding inter­actions.

## Supra­molecular features   

**Figure 2:**
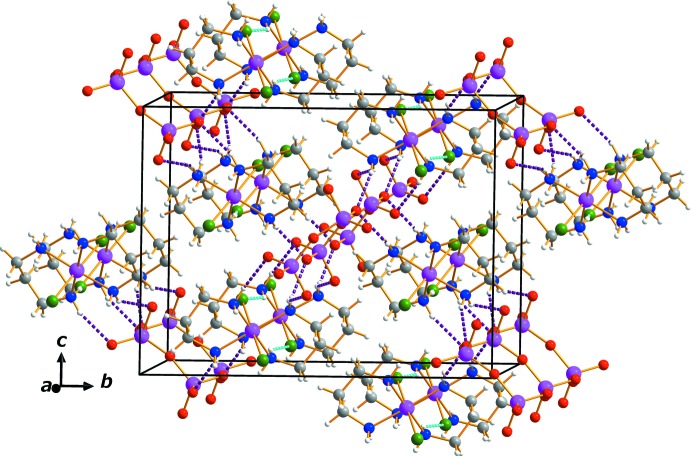
The crystal packing of complex (I), viewed along the *a*-axis direction. Dashed lines represent N—H⋯O (pink) and N—H⋯Cl (cyan) hydrogen-bonding inter­actions.

The cations and anions in the crystal structure are held tog­ether by hydrogen bonds (Table 1 ▸) between the NH_2_ donor groups of the tn ligand and Cl ligands and O atoms of the dichromate anion as acceptor groups. An extensive array of these contacts generate a three-dimensional network of mol­ecules stacked along the *a*-axis direction (Fig. 2 ▸).

## Database survey   

A search of the Cambridge Structural Database (Version 5.37, Feb 2016 with two updates; Groom *et al.*, 2016 ▸) indicates a total of 17 hits for Cr^III^ complexes containing two bidentate propane-1,3-di­amine ligands. The crystal structures of *trans*-[CrCl_2_(tn)_2_]ClO_4_ (Choi & Clegg, 2011 ▸), *trans*-[CrCl_2_(tn)_2_]_2_ZnCl_4_ (Moon *et al.*, 2012 ▸) and *trans*-[CrCl_2_(tn)_2_]_3_[Fe(CN)_6_]·6H_2_O (Kou *et al.*, 2001 ▸) have been reported previously. However, no structure of *trans*-[CrCl_2_(tn)_2_]^+^ with the [Cr_2_O_7_]^2−^ anion has been deposited.

## Synthesis and crystallization   

The free ligand propane-1,3-di­amine was obtained from Aldrich Chemical Co. and used as supplied. All other chemicals were reagent grade materials and used without further purification. As starting materials, *trans*-[CrCl_2_(tn)_2_]ClO_4_ was prepared as described in the literature (House, 1970 ▸; Choi & Clegg, 2011 ▸). The crude perchlorate salt (0.117 g) was dissolved in 10 mL of water at room temperature and added 5 mL of water containing 0.05 g of solid K_2_Cr_2_O_7_. The resulting solution was filtered and allowed to stand for two days to give green crystals of the dichromate salt suitable for X-ray structural analysis.

## Refinement   

Crystal data, data collection and structure refinement details are summarized in Table 2 ▸. All H atoms were placed in geometrically idealized positions and constrained to ride on their parent atoms, with C—H distances of 0.97 Å, and N—H distances of 0.89 Å, and with *U*_iso_(H) values of 1.2*U*_eq_ of the parent atoms. The dichromate anion is positionally disordered over two sets of sites. In a first step, the occupancies of respective pairs, O1*SA*/O1*SB*, O2*SA*/O2*SB*, O3*SA*/O3*SB* and O4*SA*/O4*SB*, were refined freely and subsequently fixed at a ratio of 0.7:0.3. The bridging atoms O1*SA*/O1*SB* sites were refined using EXYZ/EADP commands; for O3*SA*, O2*SB*, O3*SB* and O4*SB* atoms ISOR restraints were applied.

## Supplementary Material

Crystal structure: contains datablock(s) I. DOI: 10.1107/S2056989016012755/wm5308sup1.cif

Structure factors: contains datablock(s) I. DOI: 10.1107/S2056989016012755/wm5308Isup2.hkl

CCDC reference: 1498083

Additional supporting information: crystallographic information; 3D view; checkCIF report

## Figures and Tables

**Table 1:** Hydrogen-bond geometry (Å, °)

*D*—H⋯*A*	*D*—H	H⋯*A*	*D*⋯*A*	*D*—H⋯*A*
N1—H1*A*⋯O4*SA* ^i^	0.89	2.29	3.076 (4)	147
N1—H1*A*⋯O4*SB* ^i^	0.89	2.08	2.877 (14)	149
N1—H1*B*⋯O2*SA* ^ii^	0.89	2.19	3.022 (4)	156
N1—H1*B*⋯O3*SB* ^ii^	0.89	2.39	3.182 (16)	149
N2—H2*A*⋯Cl1^iii^	0.89	2.62	3.4085 (19)	149
N2—H2*B*⋯O2*SB* ^iv^	0.89	2.63	3.070 (15)	111
N3—H3*A*⋯O3*SA* ^v^	0.89	2.20	3.017 (5)	153
N3—H3*A*⋯O4*SB* ^v^	0.89	2.21	2.933 (14)	138
N3—H3*B*⋯O2*SA* ^iv^	0.89	2.28	3.027 (5)	141
N3—H3*B*⋯O2*SB* ^iv^	0.89	2.13	2.989 (16)	162
N4—H4*A*⋯O3*SA* ^i^	0.89	2.25	3.044 (4)	149
N4—H4*A*⋯O4*SB* ^i^	0.89	2.42	3.220 (14)	150
N4—H4*B*⋯Cl2^vi^	0.89	2.68	3.439 (2)	143

Symmetry codes: (i) 
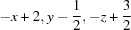
; (ii) 
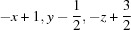
; (iii) 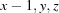; (iv) 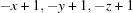; (v) 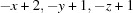; (vi) 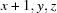.

**Table 2:** Experimental details

Crystal data	
Chemical formula	[CrCl_2_(C_3_H_10_N_2_)_2_]_2_[Cr_2_O_7_]
*M*_r_	758.32
Crystal system, space group	Monoclinic, *P*2_1_/*c*
Temperature (K)	253
*a*, *b*, *c* (Å)	6.5240 (13), 17.350 (4), 12.901 (3)
β (°)	97.18 (3)
*V* (Å^3^)	1448.8 (5)
*Z*	2
Radiation type	Synchrotron, λ = 0.610 Å
μ (mm^−1^)	1.22
Crystal size (mm)	0.13 × 0.10 × 0.09

Data collection	
Diffractometer	ADSC Q210 CCD area detector
Absorption correction	Empirical (using intensity measurements) (*HKL3000sm *SCALEPACK**; Otwinowski & Minor, 1997 ▸)
*T*_min_, *T*_max_	0.862, 0.897
No. of measured, independent and observed [*I* > 2σ(*I*)] reflections	13070, 3438, 3059
*R*_int_	0.019
(sin θ/λ)_max_ (Å^−1^)	0.667

Refinement	
*R*[*F* ^2^ > 2σ(*F* ^2^)], *wR*(*F* ^2^), *S*	0.037, 0.105, 1.10
No. of reflections	3438
No. of parameters	192
No. of restraints	24
H-atom treatment	H-atom parameters constrained
Δρ_max_, Δρ_min_ (e Å^−3^)	0.67, −0.96

Computer programs: *PAL BL2D-SMDC* (Shin *et al.*, 2016 ▸), *HKL3000sm* (Otwinowski & Minor, 1997 ▸), *SHELXT2014* (Sheldrick, 2015*a* ▸), *SHELXL2014* (Sheldrick, 2015*b* ▸), *DIAMOND 4* (Putz & Brandenburg, 2014 ▸) and *publCIF* (Westrip, 2010 ▸).

## supplementary crystallographic information

### Crystal data

**Table:**

[CrCl_2_(C_3_H_10_N_2_)_2_]_2_[Cr_2_O_7_]	*F*(000) = 776
*M**_r_* = 758.32	*D*_x_ = 1.738 Mg m^−^^3^
Monoclinic, *P*2_1_/*c*	Synchrotron radiation, λ = 0.610 Å
*a* = 6.5240 (13) Å	Cell parameters from 48108 reflections
*b* = 17.350 (4) Å	θ = 0.4–33.7°
*c* = 12.901 (3) Å	µ = 1.22 mm^−^^1^
β = 97.18 (3)°	*T* = 253 K
*V* = 1448.8 (5) Å^3^	Plate, green
*Z* = 2	0.13 × 0.10 × 0.09 mm

### Data collection

**Table:**

ADSC Q210 CCD area detector diffractometer	3059 reflections with *I* > 2σ(*I*)
Radiation source: PLSII 2D bending magnet	*R*_int_ = 0.019
ω scan	θ_max_ = 24.0°, θ_min_ = 1.7°
Absorption correction: empirical (using intensity measurements) (*HKL3000sm SCALEPACK*; Otwinowski & Minor, 1997)	*h* = −8→8
*T*_min_ = 0.862, *T*_max_ = 0.897	*k* = −23→23
13070 measured reflections	*l* = −16→16
3438 independent reflections	

### Refinement

**Table:**

Refinement on *F*^2^	Hydrogen site location: inferred from neighbouring sites
Least-squares matrix: full	H-atom parameters constrained
*R*[*F*^2^ > 2σ(*F*^2^)] = 0.037	*w* = 1/[σ^2^(*F*_o_^2^) + (0.0617*P*)^2^ + 0.9892*P*] where *P* = (*F*_o_^2^ + 2*F*_c_^2^)/3
*wR*(*F*^2^) = 0.105	(Δ/σ)_max_ = 0.001
*S* = 1.10	Δρ_max_ = 0.67 e Å^−^^3^
3438 reflections	Δρ_min_ = −0.96 e Å^−^^3^
192 parameters	Extinction correction: SHELXL2014 (Sheldrick, 2015b), Fc^*^=kFc[1+0.001xFc^2^λ^3^/sin(2θ)]^-1/4^
24 restraints	Extinction coefficient: 0.025 (3)

### Special details

**Table:**

Geometry. All esds (except the esd in the dihedral angle between two l.s. planes) are estimated using the full covariance matrix. The cell esds are taken into account individually in the estimation of esds in distances, angles and torsion angles; correlations between esds in cell parameters are only used when they are defined by crystal symmetry. An approximate (isotropic) treatment of cell esds is used for estimating esds involving l.s. planes.

### Fractional atomic coordinates and isotropic or equivalent isotropic displacement parameters (Å^2^)

**Table:**

	*x*	*y*	*z*	*U*_iso_*/*U*_eq_	Occ. (<1)
Cr1	0.49736 (4)	0.22092 (2)	0.64090 (2)	0.01917 (12)	
Cl1	0.74178 (8)	0.17096 (3)	0.54341 (5)	0.03402 (16)	
Cl2	0.26188 (9)	0.27054 (4)	0.74422 (5)	0.04056 (18)	
N1	0.4818 (3)	0.11569 (11)	0.71701 (16)	0.0333 (4)	
H1A	0.6004	0.1094	0.7584	0.040*	
H1B	0.3827	0.1194	0.7583	0.040*	
N2	0.2575 (3)	0.18582 (11)	0.52655 (15)	0.0281 (4)	
H2A	0.1386	0.1968	0.5504	0.034*	
H2B	0.2639	0.2150	0.4704	0.034*	
N3	0.5108 (4)	0.32459 (11)	0.56009 (17)	0.0355 (4)	
H3A	0.5997	0.3180	0.5138	0.043*	
H3B	0.3871	0.3320	0.5238	0.043*	
N4	0.7385 (3)	0.25541 (12)	0.75231 (14)	0.0288 (4)	
H4A	0.7266	0.2293	0.8107	0.035*	
H4B	0.8559	0.2404	0.7301	0.035*	
C1	0.4434 (4)	0.04454 (13)	0.6542 (2)	0.0386 (5)	
H1C	0.4386	0.0009	0.7008	0.046*	
H1D	0.5571	0.0364	0.6137	0.046*	
C2	0.2426 (4)	0.04817 (15)	0.5807 (2)	0.0420 (6)	
H2C	0.2113	−0.0029	0.5524	0.050*	
H2D	0.1319	0.0629	0.6204	0.050*	
C3	0.2471 (4)	0.10400 (14)	0.4914 (2)	0.0355 (5)	
H3C	0.3660	0.0928	0.4557	0.043*	
H3D	0.1241	0.0967	0.4418	0.043*	
C4	0.5687 (5)	0.39723 (14)	0.6173 (2)	0.0429 (6)	
H4C	0.4614	0.4110	0.6596	0.051*	
H4D	0.5799	0.4385	0.5676	0.051*	
C5	0.7716 (4)	0.38861 (15)	0.6867 (2)	0.0419 (6)	
H5A	0.8729	0.3674	0.6456	0.050*	
H5B	0.8194	0.4393	0.7104	0.050*	
C6	0.7610 (4)	0.33802 (15)	0.78056 (19)	0.0366 (5)	
H6A	0.8857	0.3449	0.8291	0.044*	
H6B	0.6446	0.3538	0.8156	0.044*	
Cr2	1.00840 (7)	0.57351 (2)	0.59016 (4)	0.03941 (15)	
O1SA	1.0285 (19)	0.4915 (6)	0.5165 (9)	0.064 (2)	0.35
O2SA	0.7745 (6)	0.5919 (3)	0.6061 (3)	0.0760 (12)	0.7
O3SA	1.1407 (11)	0.6446 (3)	0.5675 (4)	0.099 (2)	0.7
O4SA	1.1024 (7)	0.5391 (3)	0.7046 (3)	0.0930 (16)	0.7
O1SB	1.0285 (19)	0.4915 (6)	0.5165 (9)	0.064 (2)	0.15
O2SB	0.937 (2)	0.6545 (9)	0.5161 (12)	0.112 (4)	0.3
O3SB	0.932 (3)	0.5716 (9)	0.6899 (13)	0.118 (4)	0.3
O4SB	1.239 (2)	0.6098 (8)	0.5919 (11)	0.089 (4)	0.3

### Atomic displacement parameters (Å^2^)

**Table:**

	*U*^11^	*U*^22^	*U*^33^	*U*^12^	*U*^13^	*U*^23^
Cr1	0.01447 (17)	0.02308 (18)	0.0206 (2)	−0.00022 (10)	0.00483 (11)	0.00014 (11)
Cl1	0.0215 (3)	0.0465 (3)	0.0364 (3)	−0.0014 (2)	0.0128 (2)	−0.0111 (2)
Cl2	0.0256 (3)	0.0509 (4)	0.0486 (4)	−0.0003 (2)	0.0181 (2)	−0.0147 (3)
N1	0.0381 (11)	0.0329 (9)	0.0292 (10)	−0.0018 (8)	0.0055 (8)	0.0069 (7)
N2	0.0188 (8)	0.0314 (9)	0.0331 (10)	0.0013 (7)	−0.0005 (7)	−0.0009 (7)
N3	0.0473 (12)	0.0274 (9)	0.0316 (11)	−0.0024 (8)	0.0036 (8)	0.0026 (7)
N4	0.0233 (8)	0.0405 (10)	0.0225 (9)	−0.0040 (7)	0.0022 (6)	−0.0018 (7)
C1	0.0441 (14)	0.0248 (10)	0.0466 (15)	0.0000 (9)	0.0042 (11)	0.0077 (9)
C2	0.0361 (13)	0.0321 (12)	0.0574 (17)	−0.0112 (10)	0.0040 (11)	−0.0004 (11)
C3	0.0301 (11)	0.0350 (11)	0.0392 (13)	−0.0029 (9)	−0.0040 (9)	−0.0075 (9)
C4	0.0559 (16)	0.0247 (11)	0.0493 (16)	−0.0052 (10)	0.0117 (12)	−0.0018 (10)
C5	0.0447 (14)	0.0348 (12)	0.0488 (16)	−0.0144 (10)	0.0165 (11)	−0.0086 (10)
C6	0.0339 (12)	0.0461 (13)	0.0306 (13)	−0.0121 (10)	0.0081 (9)	−0.0142 (10)
Cr2	0.0402 (3)	0.0366 (2)	0.0431 (3)	−0.00926 (16)	0.01182 (18)	−0.01372 (16)
O1SA	0.072 (7)	0.044 (5)	0.074 (7)	0.000 (3)	0.000 (4)	−0.029 (4)
O2SA	0.052 (2)	0.108 (3)	0.069 (3)	0.031 (2)	0.0139 (17)	−0.021 (2)
O3SA	0.173 (5)	0.062 (2)	0.076 (3)	−0.078 (3)	0.074 (3)	−0.033 (2)
O4SA	0.077 (3)	0.112 (4)	0.078 (3)	−0.035 (3)	−0.039 (2)	0.023 (3)
O1SB	0.072 (7)	0.044 (5)	0.074 (7)	0.000 (3)	0.000 (4)	−0.029 (4)
O2SB	0.112 (4)	0.111 (4)	0.112 (4)	0.0009 (10)	0.0136 (11)	0.0006 (10)
O3SB	0.118 (4)	0.118 (4)	0.118 (4)	−0.0005 (10)	0.0163 (12)	−0.0006 (10)
O4SB	0.088 (4)	0.089 (4)	0.089 (4)	−0.0013 (10)	0.0110 (11)	−0.0013 (10)

### Geometric parameters (Å, º)

**Table:**

Cr1—N1	2.0814 (19)	C3—H3C	0.9700
Cr1—N4	2.0816 (19)	C3—H3D	0.9700
Cr1—N3	2.086 (2)	C4—C5	1.509 (4)
Cr1—N2	2.1020 (19)	C4—H4C	0.9700
Cr1—Cl1	2.3189 (8)	C4—H4D	0.9700
Cr1—Cl2	2.3216 (8)	C5—C6	1.504 (4)
N1—C1	1.481 (3)	C5—H5A	0.9700
N1—H1A	0.8900	C5—H5B	0.9700
N1—H1B	0.8900	C6—H6A	0.9700
N2—C3	1.489 (3)	C6—H6B	0.9700
N2—H2A	0.8900	Cr2—O3SB	1.437 (16)
N2—H2B	0.8900	Cr2—O3SA	1.554 (3)
N3—C4	1.486 (3)	Cr2—O2SA	1.597 (4)
N3—H3A	0.8900	Cr2—O4SB	1.629 (14)
N3—H3B	0.8900	Cr2—O4SA	1.639 (4)
N4—C6	1.482 (3)	Cr2—O1SB	1.725 (12)
N4—H4A	0.8900	Cr2—O1SA	1.725 (12)
N4—H4B	0.8900	Cr2—O2SB	1.729 (15)
C1—C2	1.519 (4)	Cr2—O1SB^i^	1.772 (12)
C1—H1C	0.9700	Cr2—O1SA^i^	1.772 (12)
C1—H1D	0.9700	O1SA—O1SA^i^	0.607 (12)
C2—C3	1.509 (4)	O1SA—Cr2^i^	1.772 (12)
C2—H2C	0.9700	O1SB—O1SB^i^	0.607 (12)
C2—H2D	0.9700	O1SB—Cr2^i^	1.772 (12)
			
N1—Cr1—N4	90.20 (8)	N2—C3—C2	112.6 (2)
N1—Cr1—N3	178.19 (8)	N2—C3—H3C	109.1
N4—Cr1—N3	91.25 (8)	C2—C3—H3C	109.1
N1—Cr1—N2	90.07 (8)	N2—C3—H3D	109.1
N4—Cr1—N2	179.02 (7)	C2—C3—H3D	109.1
N3—Cr1—N2	88.46 (8)	H3C—C3—H3D	107.8
N1—Cr1—Cl1	90.30 (6)	N3—C4—C5	111.1 (2)
N4—Cr1—Cl1	88.31 (6)	N3—C4—H4C	109.4
N3—Cr1—Cl1	88.66 (7)	C5—C4—H4C	109.4
N2—Cr1—Cl1	90.75 (6)	N3—C4—H4D	109.4
N1—Cr1—Cl2	88.84 (6)	C5—C4—H4D	109.4
N4—Cr1—Cl2	89.66 (6)	H4C—C4—H4D	108.0
N3—Cr1—Cl2	92.26 (7)	C6—C5—C4	114.2 (2)
N2—Cr1—Cl2	91.29 (6)	C6—C5—H5A	108.7
Cl1—Cr1—Cl2	177.79 (3)	C4—C5—H5A	108.7
C1—N1—Cr1	119.21 (15)	C6—C5—H5B	108.7
C1—N1—H1A	107.5	C4—C5—H5B	108.7
Cr1—N1—H1A	107.5	H5A—C5—H5B	107.6
C1—N1—H1B	107.5	N4—C6—C5	112.30 (19)
Cr1—N1—H1B	107.5	N4—C6—H6A	109.1
H1A—N1—H1B	107.0	C5—C6—H6A	109.1
C3—N2—Cr1	119.45 (14)	N4—C6—H6B	109.1
C3—N2—H2A	107.5	C5—C6—H6B	109.1
Cr1—N2—H2A	107.5	H6A—C6—H6B	107.9
C3—N2—H2B	107.5	O3SA—Cr2—O2SA	115.3 (3)
Cr1—N2—H2B	107.5	O3SB—Cr2—O4SB	114.8 (8)
H2A—N2—H2B	107.0	O3SA—Cr2—O4SA	107.8 (3)
C4—N3—Cr1	120.46 (17)	O2SA—Cr2—O4SA	102.3 (2)
C4—N3—H3A	107.2	O3SB—Cr2—O1SB	122.2 (8)
Cr1—N3—H3A	107.2	O4SB—Cr2—O1SB	101.1 (7)
C4—N3—H3B	107.2	O3SA—Cr2—O1SA	118.0 (6)
Cr1—N3—H3B	107.2	O2SA—Cr2—O1SA	112.0 (5)
H3A—N3—H3B	106.8	O4SA—Cr2—O1SA	98.6 (3)
C6—N4—Cr1	119.42 (15)	O3SB—Cr2—O2SB	114.5 (8)
C6—N4—H4A	107.5	O4SB—Cr2—O2SB	82.9 (7)
Cr1—N4—H4A	107.5	O1SB—Cr2—O2SB	113.6 (6)
C6—N4—H4B	107.5	O3SB—Cr2—O1SB^i^	130.7 (8)
Cr1—N4—H4B	107.5	O4SB—Cr2—O1SB^i^	107.0 (7)
H4A—N4—H4B	107.0	O1SB—Cr2—O1SB^i^	19.9 (4)
N1—C1—C2	112.4 (2)	O2SB—Cr2—O1SB^i^	95.0 (6)
N1—C1—H1C	109.1	O3SA—Cr2—O1SA^i^	112.5 (5)
C2—C1—H1C	109.1	O2SA—Cr2—O1SA^i^	100.8 (5)
N1—C1—H1D	109.1	O4SA—Cr2—O1SA^i^	117.9 (3)
C2—C1—H1D	109.1	O1SA—Cr2—O1SA^i^	19.9 (4)
H1C—C1—H1D	107.9	O1SA^i^—O1SA—Cr2	84 (2)
C3—C2—C1	113.9 (2)	O1SA^i^—O1SA—Cr2^i^	76 (2)
C3—C2—H2C	108.8	Cr2—O1SA—Cr2^i^	160.1 (4)
C1—C2—H2C	108.8	O1SB^i^—O1SB—Cr2	84 (2)
C3—C2—H2D	108.8	O1SB^i^—O1SB—Cr2^i^	76 (2)
C1—C2—H2D	108.8	Cr2—O1SB—Cr2^i^	160.1 (4)
H2C—C2—H2D	107.7		
			
Cr1—N1—C1—C2	−58.0 (3)	O3SA—Cr2—O1SA—Cr2^i^	−79 (3)
N1—C1—C2—C3	69.9 (3)	O2SA—Cr2—O1SA—Cr2^i^	59 (3)
Cr1—N2—C3—C2	55.6 (2)	O4SA—Cr2—O1SA—Cr2^i^	166 (3)
C1—C2—C3—N2	−68.5 (3)	O1SA^i^—Cr2—O1SA—Cr2^i^	−0.002 (7)
Cr1—N3—C4—C5	53.7 (3)	O3SB—Cr2—O1SB—O1SB^i^	122 (3)
N3—C4—C5—C6	−71.0 (3)	O4SB—Cr2—O1SB—O1SB^i^	−109 (3)
Cr1—N4—C6—C5	−54.5 (2)	O2SB—Cr2—O1SB—O1SB^i^	−22 (3)
C4—C5—C6—N4	72.2 (3)	O3SB—Cr2—O1SB—Cr2^i^	122 (3)
O3SA—Cr2—O1SA—O1SA^i^	−79 (3)	O4SB—Cr2—O1SB—Cr2^i^	−109 (3)
O2SA—Cr2—O1SA—O1SA^i^	59 (3)	O2SB—Cr2—O1SB—Cr2^i^	−22 (3)
O4SA—Cr2—O1SA—O1SA^i^	166 (3)	O1SB^i^—Cr2—O1SB—Cr2^i^	−0.002 (7)

Symmetry code: (i) −*x*+2, −*y*+1, −*z*+1.

### Hydrogen-bond geometry (Å, º)

**Table:**

*D*—H···*A*	*D*—H	H···*A*	*D*···*A*	*D*—H···*A*
N1—H1*A*···O4*SA*^ii^	0.89	2.29	3.076 (4)	147
N1—H1*A*···O4*SB*^ii^	0.89	2.08	2.877 (14)	149
N1—H1*B*···O2*SA*^iii^	0.89	2.19	3.022 (4)	156
N1—H1*B*···O3*SB*^iii^	0.89	2.39	3.182 (16)	149
N2—H2*A*···Cl1^iv^	0.89	2.62	3.4085 (19)	149
N2—H2*B*···O2*SB*^v^	0.89	2.63	3.070 (15)	111
N3—H3*A*···O3*SA*^i^	0.89	2.20	3.017 (5)	153
N3—H3*A*···O4*SB*^i^	0.89	2.21	2.933 (14)	138
N3—H3*B*···O2*SA*^v^	0.89	2.28	3.027 (5)	141
N3—H3*B*···O2*SB*^v^	0.89	2.13	2.989 (16)	162
N4—H4*A*···O3*SA*^ii^	0.89	2.25	3.044 (4)	149
N4—H4*A*···O4*SB*^ii^	0.89	2.42	3.220 (14)	150
N4—H4*B*···Cl2^vi^	0.89	2.68	3.439 (2)	143

Symmetry codes: (i) −*x*+2, −*y*+1, −*z*+1; (ii) −*x*+2, *y*−1/2, −*z*+3/2; (iii) −*x*+1, *y*−1/2, −*z*+3/2; (iv) *x*−1, *y*, *z*; (v) −*x*+1, −*y*+1, −*z*+1; (vi) *x*+1, *y*, *z*.
